# Does Resting Motor Threshold Predict Motor Hand Recovery After Stroke?

**DOI:** 10.3389/fneur.2018.01020

**Published:** 2018-11-29

**Authors:** Charlotte Rosso, Jean-Charles Lamy

**Affiliations:** ^1^Institut du Cerveau et de la Moelle épinière, ICM, Inserm U 1127, CNRS UMR 7225, Sorbonne Université, Paris, France; ^2^APHP, Urgences Cérébro-Vasculaires, Hôpital Pitié-Salpêtrière, Paris, France

**Keywords:** stroke, Transcranial Magnetic Stimulation, corticopinal excitability, motor function, outcome

## Abstract

**Background:** Resting Motor threshold (rMT) is one of the measurement obtained by Transcranial Magnetic Stimulation (TMS) that reflects corticospinal excitability. As a functional marker of the corticospinal pathway, the question arises whether rMT is a suitable biomarker for predicting post-stroke upper limb function. To that aim, we conducted a systematic review of relevant studies that investigated the clinical significance of rMT in stroke survivors by using correlations between upper limb motor scores and rMT.

**Methods:** Studies that reported correlations between upper limb motor function and rMT as a measure of corticospinal excitability in distal arm muscle were identified via a literature search in stroke patients. Two authors extracted the data using a home-made specific form. Subgroup analyses were carried out with patients classified with respect to time post-stroke onset (early *vs*. chronic stage) and stroke location (cortical, subcortical, or cortico-subcortical). Methodological quality of the study was also evaluated by a published checklist.

**Results:** Eighteen studies with 22 groups (*n* = 508 stroke patients) were included in this systematic review. Mean methodological quality score was 14.75/24. rMT was often correlated with motor function or hand dexterity (*n* = 15/22, 68%), explaining on average 31% of the variance of the motor score. Moreover, the results did not seem impacted if patients were examined at the early or chronic stages of stroke. Two findings could not be properly interpreted: (i) the fact that the rMT is an independent predictor of motor function as several confounding factors are well-established, and, (ii) whether the stroke location impacts this prediction.

**Conclusion:** Most of the studies found a correlation between rMT and upper limb motor function after stroke. However, it is still unclear if rMT is an independent predictor of upper limb motor function when taking into account for age, time post stroke onset and level of corticospinal tract damage as confounding factors. Clear-cut conclusions could not be drawn at that time but our results suggest that rMT could be a suitable candidate although future investigations are needed.

**Systematic Review Registration Number:** (https://www.crd.york.ac.uk/prospero/): ID 114317.

## Introduction

Upper limb motor function, and more specifically hand motor function is difficult to predict in stroke survivors. Although recent clinical papers pointed out the predictive value of the proportional recovery rule, meaning that patients will recover about 70% of the lost function, this rule is seriously challenged by the prediction of the most severe ones (1, 2) in whom different profiles of recovery ranging from nearly no improvement to tremendous one have been observed. Indeed, researchers and clinicians are still struggling to explain these different patterns of recovery. In this context, developing and implementing biomarkers in stroke recovery research is more than ever challenging (3, 4). As regards post-stroke upper limb motor function, corticospinal excitability measured by Transcranial Magnetic Stimulation (TMS) has been identified as a possible biomarker. Some reviews already attempted to define the predictive value of TMS-induced Motor Evoked Potentials (MEPs) in arm motor function (5, 6). In contrast, resting Motor Threshold (rMT), i.e., the minimum amount of energy necessary to evoke a MEP in the relaxed target muscle has been less studied. In this paper, we review the clinical significance of corticospinal excitability, using rMT and not the MEPs amplitude, in stroke patients. This work is divided into two parts. The first one deals with the general principles of measuring rMT, its variability and values in stroke. The second part is a systematic review of relevant studies that investigated the clinical significance of rMT in stroke by the means of correlations between upper limb motor scores and rMT.

## Resting motor threshold as a measure of corticospinal excitability

### Definition

According to the International Federation of Clinical Neurophysiology (IFCN), rMT is defined as the lowest stimulus intensity (expressed as a percentage of maximal stimulator output-MSO) required to induce a MEP with a peak-to-peak amplitude of at least 50 microvolts in 5 out of 10 consecutive trials in the relaxed target muscle (7).

The motor threshold depends on the excitability of several neural elements, which are excited by TMS and propagate the elicited action potential including the cortico-cortical axons, their excitatory synaptic contacts with the corticospinal neurons, the initial axon segments of the corticospinal neurons (8) but also the spinal cord structures (9, 10).

### MT variability and influential factors

Both intra (between repeated stimulation sessions within the same subject) and inter-individual variability (between-subjects) of TMS-induced MEP are well-known and contribute to the overall heterogeneity of the measurement (11).

For inter-individual variability, one critical factor is the coil-to-cortex distance (12, 13). When targeting the hand motor area, the coil-to-cortex distance is defined as the shortest distance between the scalp and the hand knob area of the primary motor cortex and is critical in determining the amount of energy required to depolarize the corticospinal tract (CST).

The role of age is still a matter of debate. Whereas, it has been documented that rMT decreases with age (14) a recent meta-analysis reports the opposite effect, i.e., increased rMT with age (15). Among the possible other factors of inter-individual variability, drugs intake are of importance. Indeed, Voltage-Gate Sodium Channels antiepileptic drugs, i.e., carbamazepine, phenytoin, and lamotrigine increase rMT, i.e., these drugs reduce CST excitability. In contrast, ketamine, an N-methyl-D-aspartate (NMDA) receptor antagonist that indirectly facilitates glutamate neurotransmission dose-dependently decreases rMT, i.e., it increases CST excitability (16).

Intra-individual variability corresponds to the intrinsic fluctuations of the excitability of cortical and spinal neurons that cause trial-to-trial variability in MEP amplitude (17). While physiological noise introduces some uncertainties and cannot be eliminated (18), other technical and physiological variables should be kept constant during rMT measurements such as the level of arousal or the time of the session during the day (rMT being sensitive to the nycthemeral cycle). From a technical point of view, the type and size of the coil have to be kept constant. Thus, smaller coils give higher rMT, as well as circular coils *vs*. figure-of-eight shape coils (17). Coil orientation (delivering posterior-anterior, lateromedial, anteroposterior currents), pulse waveform (i.e., monophasic or biphasic) and type of stimulators are also known to affect the rMT (19, 20).

However, when these factors are controlled, the intraclass coefficient of the intra-individual variability of rMT is good (21).

### Impact of stroke on MT

Stroke affects corticospinal excitability and, as a result, the rMT. A recent review summarized the neurophysiological effects of stroke on rMT [see (22) for further details]. Briefly, the rMT is higher in the affected hemisphere when compared to the unaffected one or to healthy subjects. The exact time course of rMT after stroke is not well-known. It probably reduces over time after stroke but remains higher in the affected hemisphere (AH) with respect to the unaffected hemisphere (UH) at the chronic stage. For the UH, the meta-analysis of McDonnell et al. (22) found no differences in rMT when compared to healthy controls (22 studies, 821 participants), regardless of the stage of stroke (i.e., early or chronic).

## rMT as a biomarker of stroke hand function

### Definition of a biomarker

According to a recent consensus paper (3, 4), a stroke recovery biomarker can be defined as “an indicator of disease state that can be used as a measure of underlying molecular/cellular processes that may be difficult to measure directly in humans.”

A biomarker could be used (i) to understand outcome/impairment or, (ii) to predict a future outcome or recovery (defined as the change in the clinical score) or a treatment response. We propose to review whether rMT, measured by TMS, can be considered as a biomarker according to this definition.

### Systematic review of the literature

#### Aims

The overall goal of this review is to determine whether rMT can act as a biomarker that could (i) understand impairment (UI), (ii) predict the outcome (PO), and (iii) predict recovery (PR) of the distal upper limb motor function after stroke. We did not focus on treatment response. We defined a study as UI if the measure of rMT and the clinical scores were obtained at the same time point. We defined PO if the rMT was measured at T1 and scores were obtained later (T2), and PR, if rMT was collected at T1 and motor scores at T1 and T2 (i.e., PR represents the changes in the motor scores between T2 and T1).

#### Methods

PRISMA and PICOS checklists are available in the Supplementary Material available on line. This systematic review has been registered to PROSPERO (https://www.crd.york.ac.uk/prospero/), ID 114317.

##### Search strategy

The search strategy was formulated in broader terms voluntarily, in order to ensure exhaustivity. The Mesh terms “transcranial magnetic stimulation” AND “stroke” were combined. We searched the following databases from inception until June 2018: Medline and EMBASE. The language was restricted to English. The number of articles corresponding to these Mesh terms was 1798.

Studies were then included if (i) TMS was used to investigate ipsilesional rMT in participants with a confirmed diagnosis of stroke of any type, with or without comparison to healthy controls, (ii) rMT was collected in hand or forearms muscles (if rMT was recorded from multiple muscle groups, only the distal arm muscle data were included), (iii) motor upper limb or hand function was evaluated at the time of the TMS session or later and, (iv) individual patient data (with rMT and motor scores) were available even though the primary aim of the publication was not to investigate rMT but rather other TMS parameters.

Studies were excluded if (i) rMT was recorded from the proximal arm muscles (i.e., biceps brachii) or from lower limb/pharyngeal/trunk muscles, (ii) motor threshold was collected under active condition (i.e., during a contraction of the target muscle), (iii) rMT was collected after an intervention (i.e., novel rehabilitation techniques, after non-invasive brain stimulation such as repetitive TMS…) and, (iv) the sample size was less than 5 patients, including case reports.

Two researchers ran each database search independently and then compared findings. Search results duplicates were removed. The same two researchers screened the search findings for eligibility, using article titles and abstracts, for the inclusion of appropriate participants, and measurements. When it was unclear if the study met all of the inclusion criteria on the initial title/abstract screening, the full text was obtained and assessed for eligibility.

##### Data extraction and management

One author extracted data from the included studies using a standardized data extraction form specifically designed for this review. Extracted data included the following information from the methods section of the articles: aim of the study (UI, PO, PR), detailed description of the participants (age, sex, type and location of stroke, time since post-stroke onset, motor scores), research methods (type and size of the coil, target muscle) and type of the motor score. The correlation coefficient between rMT and motor scores, the R2 and the statistical significance were recorded when available.

##### Subgroup analyses

We planned *a priori* subgroup analyses to compare results from (i) acute (within 7 days) *vs*. subacute (within 3 months after stroke onset) *vs*. chronic phase (more than 3 months) and, (ii) the location of stroke (subcortical *vs*. cortical *vs*. cortico-subcortical).

##### Risk of bias

*Risk of individual bias: methodological quality assessment*. We extracted information on the methodological quality of each study included in our systematic review. For this methodology quality assessment, two reviewers independently assessed the quality of each study using the checklist designed by Chipchase et al. (23) for TMS studies. This checklist was modified, as in McDonnell et al. (22). Four items were removed because they related to paired-pulse TMS paradigms, an additional one because it dealt with healthy participants and a last one because it assessed repetitive sessions within the same subjects. As a result, a total quality score of 24 was obtained. We coded the studies as low (score > 16), unclear (scores ranging from 9–16) or high risk (score ≤ 8) of bias.

*Risk of bias inherent to group analysis*. We considered all potential sources of bias in the conduct of our systematic review, such as recruitment bias, publication bias and selection bias.

#### Results

##### Descriptions of the included studies (figure 1, tables 1, 2)

**Figure 1:**
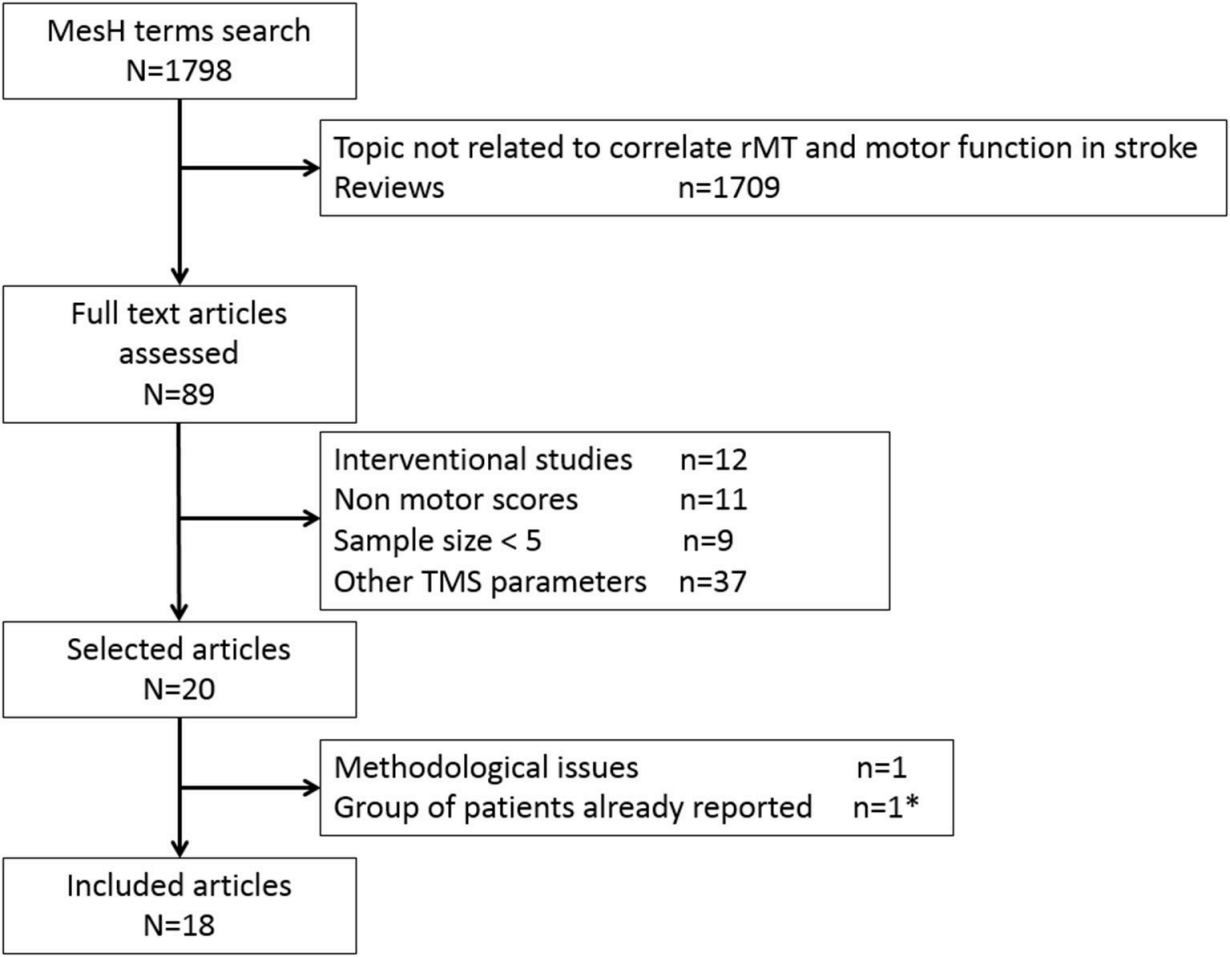
Flow chart of the systematic review process. * Ward et al. (24) was excluded to keep the more recent study (25).

Of the 20 studies included for analysis (Flow chart: Figure 1), two studies reported the same stroke patients (24, 25), so the more recent study was selected (25). One study (43) was discarded because of methodological issues (no information about rMT definition and TMS equipment used for recording was provided). Eighteen studies were included depicting a total of 508 stroke patients. Two studies reported separated groups in the main text: the first one (27) reported two groups (subacute vs. chronic) and the second one (33) four groups according to the infarct location. As a result, 22 samples from 18 studies were included in this review (25–42). We further referenced throughout the following as a number of samples and not studies for clarity. Among these 22 samples, 20 samples reported the correlation between rMT and motor scores in the main text. For two of them, we computed correlation, based on the individual patient data, using Spearman rank coefficients.

**Table 1:** Clinical characteristics of the patients included.

**Study**	***N***	**Purpose**	**I/H**	**Location SC/C/SCS/BS**	**TPSO(months)**	**Stage**	**Age**	**Motor scores**	**Lost FU**
Bastings et al. (26)^ch^	12	PI	12/0	6/0/6/0	14	C	na	Frenchay	
Brouwer et al. (27)^su^	14	PI	9/5	7/0/7/0	96	C	62	Tapping,MVC	
Brouwer et al. (27)	14	PI	11/3	6/0/8/0	1.4	S	67	Tapping, MVC	
Borich et al. (28)	36	PI	36/0	36/0/0/0	na	C	65	BBT	
Cakar et al. (29)	22	PI	22/0	3/10/9/0	na	C	64	Tapping, Brunnstom	
Freundlieb et al. (30)	12	PO	12/0	6/3/1/0	0.08	A	68	FM, JTI, 9HPT	2
Huynh et al. (31)	31	PO	na	17/14/0/0	0.2	A	64	FM	14
Jo et al. (32)	113^**^	PO	84/29^**^	75/21/0/17^**^	0.4^**^	S	58	FM	0
Liepert et al. (33)^a^	7	PI	na	0/7/0/0	na	S	73	GS, 9HPT	
Liepert et al. (33)^b^	13	PI	na	13/0/0/0	na	S	67	GS, 9HPT	
Liepert et al. (33)^c^	13	PI	na	13/0/0/0	na	S	63	GS, 9HPT	
Liepert et al. (33)^d^	10	PI	na	0/0/0/10	na	S	71	GS, 9HPT	
Pennisi et al. (34)	40	PI	40/0	40/0/0/0	na	C	64	MRC, 9HPT	
Shiner et al. (35)	9	PI	6/3	na	17	C	54	BBT, GS, FM	
Simis et al. (36)	35	PI	na	10/23/0/2	15	C	62	FM	
Stinear et al. (37)	46	PR	46/0	32/2/7/5	0.43	S	67	FM, ARAT	0
Takechi et al. (38)	24	PI/PO	10/14	24/0/0/0		S	64	FM,JTI, GS	
Takeuchi et al. (38)	38	PI	na	18/20/0/0	50	C	62	FM	
Thibaut et al. (39)	55	PI	49/6	na	31	C	62	FM	
Veldema et al. (40)	18	PI/PR	18/0	6/3/6/1	1.7	S	70	ARAT, WMFT	9
Ward et al. (25)	9	PI	na	8/0/0/0	11.5	C	48	9HPT	
Swayne et al. (41)	10	PI	10/0	5/1/3/0	na	S	58	9HPT	

** *Of the 113 patients, MEPs were elicited only in 40 patients (only them were used for correlation)*.

*N, number; I, ischemic stroke; H, hemorrhagic stroke; SC, subcortical; C, cortical; CSC, cortico-subcortical; BS, Brainstem; TPSO, time post-stroke onset; C, chronic; A, acute; S, subacute; PI, predict impairment; PO, predict outcome; PR, predict recovery; na, not available; BBT, Box and block test; FM, Fugl Meyer; GS, grip strength; MVC: maximal voluntary contraction; ARAT, Action Research Arm Test; 9HPT, 9-hole peg test; JTT, Jebsen Taylor test; WMFT, Wolf motor function test; FU, follow-up*.

a *group of patients with cortical lesions*.

b *group of patients with basal ganglia lesions*.

c *group pf patients with internal capsule lesions*.

d *group of patients with brainstem lesions*.

ch: chronic stage group of Brouwers et al. (27); su: subacute stage group of Brouwers et al. (27).

**Table 2:** TMS measurements characteristics.

**Study**	**Muscle**	**Type coil**	**Size coil**	**MEP size for determining rmt (microvolts)**	**Absence of MEP at T1**	**rMT AH**	**rMT UH**	**Imputation**
Bastings et al. (26)	FDI	8-Coil	na	na	3 (25%)	70	67	Yes
Brouwer et al. (27) ch	FDI	8-Coil	80	50	2 (14%)	76	63	No
Brouwer et al. (27) su	FDI	8-Coil	80	50	3 (21%)	85	63	No
Borich et al. (28)	ECR	8-Coil	70	na	2 (6%)	43	41	No
Cakar et al. (29)	ADM	parabolic	na	50	na	50	37	No
Freundlieb et al. (30)	FDI	na	na	na	3 (25%)	35	38	No
Huynh et al. (31)	APB	circular	90	200	6 (19%)	66	58	Yes
Jo et al. (32)	FDI	8-Coil	70	50	73 (65%)^**^	51	na	No
Liepert et al. (33)^a^	FDI	8-Coil	na	50	na	56	46	na
Liepert et al. (33)^b^	FDI	8-Coil	na	50	na	55	44	na
Liepert et al. (33)^c^	FDI	8-Coil	na	50	na	50	45	na
Liepert et al. (33)^d^	FDI	8-Coil	na	50	na	59	45	na
Pennisi et al. (34)	FDI	circular	90	20	0 (0%)	48	42	No need
Shiner et al. (35)	FDI	circular	125	50	4 (44%)	na	na	Yes
Simis et al. (36)	FDI	na	na	50	3 (9%)^**^	na	na	No need
Stinear et al. (37)	ECR	8-Coil	70	70	10 (22%)	71	45	Yes
Takechi et al. (38)	FDI	8-Coil	90	50	10 (42%)	74	47	No
Takeuchi et al. (42)	FDI	8-Coil	70	50	20 (53%)^**^	52	52	No
Thibault et al. (39)	FDI	8-Coil	70	50	3 (5%)	C1: 59. C2 : 73	C1 : 52. C2 : 55	No
Veldema et al. (40)	APB	8-Coil	70	50	10 (56%)	86	64	Yes
Ward et al. (25)^b^	FDI	8-Coil	70	50	0 (0%)	58	Na	No need
Swayne et al. (41)	FDI	8-Coil	70	50	0 (0%)	64	42	Yes

** *(excluded from analysis)*.

*FDI, First Digital Interosseus; APB, Abductor Pollicis Brevis; ADM, Abductor Digiti Minimi; Na, not available; C1, center 1; C2, center 2; MEP, motor evoked potential; rMT, resting motor threshold; AH, affected hemisphere; UH, unaffected hemisphere*.

a *group of patients with cortical lesions*.

b *group of patients with basal ganglia lesions*.

c *group pf patients with internal capsule lesions*.

d *group of patients with brainstem lesions; ch: chronic stage group of Brouwers et al. (27); su, subacute stage group of Brouwers et al. (27). Imputation, imputation of MT in patients without MEPs*.

The purpose of identifying samples was to understand impairment (UI–*n* = 18), predict outcome (PO–*n* = 4), and predict recovery (PR–*n* = 2). Two samples investigated both UI and PO or UI and PR.

**Figure 2:**
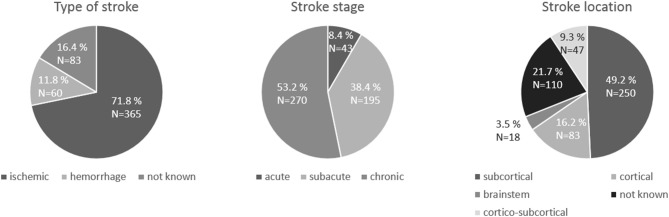
Pie charts of the characteristics of the stroke patients: stroke type, stroke stage and stroke location.

The clinical characteristics of stroke patients are displayed in Figure 2. As regards TMS measurements (Table 2), First Dorsal Interosseus (FDI) was the distal muscle recorded in 17 samples, Extensor Carpis Radialis (ECR) in 2, Abductor Pollicis Brevis (APB) in 2 and Abductor Digiti Minimi (ADM) in 1 sample. Motor function and hand dexterity were assessed using several clinical scores (see Table 1).

##### Correlations between motor distal upper limb function and rMT

Mean rMT was 61 ± 13%MSO (SD) on the AH and 51 ± 9%MSO on the UH. Seventeen samples reported that it was not possible to elicit MEP on the AH (mean proportion of the patients without MEPs: 24 ± 21%). When MEPs could not be evoked, so that rMT could not be determined, authors assigned to rMT an arbitrary value of 100% MSO in 4 samples, 110% in one sample and 120% in one sample. In some samples (*n* = 3), patients without MEP on the AH were excluded from the analysis.

Fifteen samples found a significant correlation between motor scores and rMT, with R2 ranging from 12 to 64% (mean: 31%) whereas 7 samples found no significant correlations. Regardless of the value of the correlation, the fact that rMT was an independent predictor of motor function is of importance. This point was raised in only four samples (*n* = 75), by adjusting the model according to well-known confounding factors (i.e., age, time post stroke onset or other TMS parameters) but the correlation between motor scores and rMT remained significant.

##### Subgroup analyses

*Early vs. chronic patients*. We divided our samples into three categories: acute (< 7days), subacute (7 days to 3 months) and chronic (>3 months). Table 3 displays the values of rMT, the proportion of patients without MEP and the correlations between motor scores and rMT. It is worth noting that results dealing with the acute period must be taken with caution given: (i) the small number of both samples (*n* = 2) and patients (*n* = 43), and (ii) the possible bias in the recruitment of these acute patients (who are likely less severe to be able to handle TMS measurements). For these reasons, we focused the analysis on the comparison of subacute *vs*. chronic stroke patients. On the AH, rMT was higher at the subacute *vs*. chronic phase but the difference did not reach significance (p: 0.15). On the UH, rMT was unchanged. The proportion of patients who did not exhibit MEP decreased from 34 to 17% (*p* < 0.001) between the subacute *vs*. chronic stage, suggesting that some MEPs might reappear during recovery. When rMT correlated to motor scores, the proportion of variance of the motor score explained by the rMT was around 30% in both stages.

**Table 3:** Resting motor threshold and correlation with clinical score with respect to time post-stroke onset.

	**Acute *N* = 43**	**Subacute *N* = 195**	**Chronic *N* = 270**	***P-*value S *vs*. C**
rMT AH (% MSO)	51 ± 22	65 ± 13	58 ± 13	0.15
rMT UH (% MSO)	48 ± 14	49 ± 8	50 ± 11	0.90
MEP– (%)	22 ± 4	34 ± 24	17 ± 20	< 0.001
Samples with non-significant correlations	2/2	3/10	2/10	0.62
	(100%)	(30%)	(20%)
R2 (in samples with significant correlations)	–	30 ± 14	32 ± 18	0.69

*S, Subacute; C, chronic; AH, affected hemisphere; UH, unaffected hemisphere; MEP-, patients in whom it was not possible to elicit MEP; MSO, Maximal stimulator output*.

*Location of stroke*. Samples reporting either individual patient data (25, 26, 40) or samples with location subgroups analyses (33) were analyzed in order to investigate whether stroke location (cortical-C, cortico-subcortical-CSC, subcortical-SC) impacts rMT values or its correlation with motor scores. As reported in Table 4, the results were quite heterogeneous, with no clear pattern indicating that rMT values relate to a specific location of stroke. In all these groups (C, CSC, SC), rMT in the AH was higher than in the UH. Although Liepert et al. (33) reported that the correlation between rMT and motor scores was only present in lesions involving the corticospinal tract at the subcortical level (internal capsule and pons) and not at the cortical level, these results were not confirmed by others. Indeed, Jo et al. (32) reveal no significant difference between each lesion site with respect to the stroke location classified as cortical, subcortical and brainstem. Overall, it is not possible to draw any conclusion on the potential impact of stroke location on rMT predictive value or its correlation with motor scores.

**Table 4:** TMS characteristics of the four studies (seven samples) examining the impact of location on the correlation between rMT and motor scores.

	**C *N* = 11**	**SC *N* = 46**	**CSC *N* = 12**	**BS *N* = 12**
**rMT AH (%MSO)**				
Liepert et al. (33)	56 ± 12	53 ± 12	_	59 ± 11
Ward et al. (25)	_	57 ± 19	_	53 ± 2
Bastings et al. (26)	_	73 ± 17	82 ± 26	_
Veldema et al. (40)	96 ± 9	89 ± 19	78 ± 19	_
**rMT UH (%MSO)**				
Liepert et al. (33)	46 ± 6	45 ± 10	_	45 ± 8
Ward et al. (25)	_	_	_	_
Bastings et al. (26)	_	67 ± 12	68 ± 23	_
Veldema et al. (40)	54 ± 7	71 ± 19	60 ± 7	_

*C, cortical; SC, subcortical; CSC, corticosubcortical; BS, brainstem; AH, affected hemisphere; UH, unaffected hemisphere; MSO, Maximal Stimulator Output*.

##### Risk of bias

*Risk of individual bias: methodological quality assessment*. All studies were assessed using the checklist designed by Chipchase et al. (23). The average quality score was 14.75 (SD: 2.53, ranging from 9 to 19). Two studies scored less than half the total score (i.e., 12) (36, 39). Five studies had a low risk (28%), 13 were unclear (72%) and none was rated with a high risk.

*Risk of bias inherent to group analysis*. As regards recruitment bias, all studies included patients with first-ever stroke with motor impairments. However, some of these added more inclusion criteria, especially for the type of stroke [i.e., lacunar in Pennisi et al. (34)] or for the severity of the motor deficits [at least 10 degrees of wrist extension for Simis et al. (36) and Thibaut et al. (39)]. These more stringent criteria could limit the extrapolation of these results. Publication bias may be caused, at least in part, by journal editors and reviewers who are more likely to accept studies with statistically significant results. Finally, there are others (methodological) biases given that confounding factors have not been taken into consideration.

#### Discussion

This systematic review provided two main findings. First, rMT often correlated with motor function or hand dexterity. Second, the results did not seem impacted by the duration of the disease (i.e., early or chronic stages). Two findings could not be properly interpreted: (i) the fact that rMT is an independent predictor of motor function given that several confounding factors are well-known and, (ii) whether the stroke location impacts this prediction.

##### MT as a predictor of upper limb motor function

Fifteen samples (68%) found a correlation between upper limb motor function and MT, wherein four of these confirmed it was an independent predictor using regression analysis. Among the seven samples in which no correlation was found to be significant, two were collected at the acute stage (< 7 days).

rMT corresponds to the threshold where the pyramidal tract responds to the magnetic stimulus. However, the basic neurophysiology of rMT is incompletely understood regarding the generation of transmembrane excitation and is still a matter of debate (44). The hypothesis that could explain why rMT is correlated to motor function could be that it integrates many pieces of information about the structural and the functional integrity of the motor system. One current and a somehow logical statement is that rMT reflects the properties of the corticospinal tract. In one study (45), rMT was independently explained (R2: 13%) by the radial diffusivity in the internal capsule, suggesting that the coherence of the fiber orientation determines the intensity needed to produce a MEP. rMT has been shown to be correlated with the white matter properties of the premotor, motor, and prefrontal regions, supporting the hypothesis that fractional anisotropy is a surrogate marker of the organization of the cortico-cortical connections that may facilitate the depolarization of the primary motor cortex (M1) cells (46).

Second, as rMT reflects the neuronal membrane excitability, it strongly relates to the orientation and structure of the pyramidal cells within M1. Indeed, modeling studies have shown that individual cortical anatomy has a major impact on TMS-induced electrical field distributions (47–49). It has also been demonstrated that field strength significantly enhanced when currents run approximately perpendicular to the local orientation of the gyri (17, 50).

However, rMT could depend, not only on the neuronal membrane excitability by itself but also on the interactions of the vicinity on these cells (premotor and somatosensory cortices) that could modify the state of excitability. This statement is reinforced by the fact that TMS suffers from a poor spatial resolution. Using Dynamical Causal Modeling, an MRI technique that allows making inference between regions during a task, Sarfeld et al. (51) demonstrated that the higher the excitability of left M1 the stronger the coupling between left supplementary motor area and M1. In line with these results, we demonstrated in a previous study (52) that rMT was in part explained by the functional connectivity of the premotor cortex and M1. These results underlined the major role of the premotor areas and the cortico-cortical connections toward M1 in the excitation of the CST fibers (through trans-synaptic pathways).

Finally, if rMT integrates the information from M1 itself, and from the surrounding regions at the cortical level, it is also susceptible to synaptic influences at the spinal level. The corticomotoneuronal pathway is a disynaptic route where the first neuron makes its junction spinal motoneurons. Obviously, MEPs are influenced not only by the excitability of the corticospinal cells but also by the excitability of the spinal motoneurons to which they project (9, 10). It represents the sum of the events at all these synapses as well as the spinal postsynaptic excitability. Overall, this determines whether corticospinal cells are activated and synchronized.

Together, these suggest that altered rMT could relate to motor function outcomes.

To summarize, our results support the view that rMT could be a suitable biomarker of post-stroke motor function as it responds to the definition recently published as “an indicator of disease state that can be used as a measure of underlying molecular/cellular processes that may be difficult to measure directly in humans.” This statement applies for the subacute and the chronic phase. This conclusion cannot be extrapolated to the acute phase, where the sample sizes were too small.

##### MT and stroke location

We could not draw a meaningful conclusion about whether location of stroke influences or not the association between rMT and motor outcome. If most of the samples reported stroke locations, only few of them performed subgroup analyses between cortical, subcortical and cortico-subcortical lesions.

Liepert et al. (33) reported a significant association between rMT and motor function only in lesions involving the CST at the subcortical level. This was explained by the fact that rMT was significantly higher in subcortical lesions whereas it did not differ with respect to the UH for lesions encompassing M1 or the basal ganglia. These results were supported by others. According to Freundlieb et al. (30), purely subcortical lesions are more likely to globally disrupt efferent motor pathways and thereby to raise rMTs. This could be explained by the susceptibility to ischemia which could differ for low *vs*. high rMT pyramidal cells. There are also some reports in which (at least in the early post-stroke phase) rMT is higher in patients with subcortical compared to cortical ones (53, 54). Indeed, Delvaux et al. (55) reported near normal rMT in a group of patients studied the first day after a mainly cortical stroke (55). It may be that a subcortical lesion damaging a large number of densely packed fibers can compromise responsiveness to TMS more than a cortical lesion that often damages patchy areas of survived tissue. However, Catano et al. (56) found no clear association during the first 3 months post-stroke between rMT and lesion location (56). As this latter, when we reported rMT from the three other samples that provided individual patients data and allow us to analyse the rMT according to stroke location, we could not find a clear pattern of high rMT for subcortical and normal rMT for cortical strokes.

##### Limitations

As in all systematic review, and especially those who include studies with small sample sizes, our results should be taken with caution mainly because of methodological purposes. For example, from a technical perspective, most of our samples used 70 mm 8-shape coils but some used coils of different shapes and sizes that could influence the absolute value of the rMT. The definition of rMT was relatively homogeneous and in accordance with the IFCN definition (7) except in Huynh et al. (31). The number of trials was 5 out of 10 in 19 samples (86%). Second, the lack of individual patients data reported hampered us for more advanced statistics, and further analyses. Only four studies reported data for each patient for a total of 76 patients. Finally, the type of motor scores was quite heterogeneous. Some of them measured gross motor function (such as the Fugl-Meyer) while others measured fine dexterity (i.e., finger tapping, 9HPT) (57). We were not able to perform subgroups analysis according to gross or fine motor function assessment because of the small sample of studies included.

## Conclusion

The results of this systematic review support the need for future work regarding the rMT as a potential biomarker of post-stroke upper limb motor function. Most of the studies found a correlation between rMT and clinical scores. However, it is still unclear if rMT is an independent predictor of upper limb motor function when taking into account for age, time post-stroke onset and level of CST damage as the main confounding factors. Clear-cut conclusions could not be drawn at that time but our results suggest that rMT could be a suitable candidate although future investigations are needed.

## Author contributions

All authors listed have made a substantial, direct and intellectual contribution to the work, and approved it for publication.

### Conflict of interest statement

The authors declare that the research was conducted in the absence of any commercial or financial relationships that could be construed as a potential conflict of interest.
