# Supplementary material for: Does Resting Motor Threshold Predict Motor Hand Recovery After Stroke?
Source: Front Neurol. 2018 Nov 29;9:1020. doi: 10.3389/fneur.2018.01020 (PMC6281982; doi:10.3389/fneur.2018.01020)
Supplement: Supplementary file 1 [file Table_1.DOCX]

**PICO Worksheet and Search Strategy Protocol**

**1. Define your question using PICO by identifying: Patient/Problem, Intervention, Comparison group and Outcome:**

Patient/Problem : Stroke patients with upper limb distal motor deficits

Intervention Not applicable

Comparison_Not applicable

Outcome : Correlation between resting motor threshold and upper limb distal motor deficits in stroke patients

Write out your question: Does resting motor threshold predict hand motor recovery after stroke ?

**2. Type of question/problem:** Prognosis

**3. Type of studies/publications to include in the search: Check all that apply:**

- □  Meta-analysis
- □  Systematic review
- □  Clinical practice guidelines
- □  Research report or other grey literature

□ Randomized controlled trial

X Research studies or articles

□ Case report or series

**4. List main topics and alternate terms from your PICO question that can be used for your search:**

Corticospinal excitability ; stroke ; motor ; resting motor threshold ; transcranial magnetic stimulation.

- **5. Write out your search strategy:**
- “transcranial magnetic stimulation” AND “stroke”

1. **List any limits that may apply to your search:**

Gender: no limit

Age: no limit

Year(s) of publication: no lower limit-2018

Language(s): english

1. **List the databases you will search:**

Medline

EMBASE

This form is adapted from: Miller, S.A. (2001). *PICO worksheet and search strategy.* US National Center for Dental Hygiene Research.
